# Effector and Naturally Occurring Regulatory T Cells Display No Abnormalities in Activation Induced Cell Death in NOD Mice

**DOI:** 10.1371/journal.pone.0021630

**Published:** 2011-06-27

**Authors:** Ayelet Kaminitz, Esma S. Yolcu, Enosh M. Askenasy, Jerry Stein, Isaac Yaniv, Haval Shirwan, Nadir Askenasy

**Affiliations:** 1 Frankel Laboratory, Center for Stem Cell Research, Schneider Children's Medical Center of Israel, Petach Tikva, Israel; 2 Sackler School of Medicine, Tel Aviv University, Tel Aviv, Israel; 3 Institute for Cellular Therapeutics and Department of Microbiology and Immunology, University of Louisville, Louisville, Kentucky, United States of America; 4 Bone Marrow Transplant Unit, Schneider Children's Medical Center of Israel, Petach Tikva, Israel; 5 Department of Pediatric Hematology-Oncology, Schneider Children's Medical Center of Israel, Petach Tikva, Israel; La Jolla Institute of Allergy and Immunology, United States of America

## Abstract

**Background:**

Disturbed peripheral negative regulation might contribute to evolution of autoimmune insulitis in type 1 diabetes. This study evaluates the sensitivity of naïve/effector (Teff) and regulatory T cells (Treg) to activation-induced cell death mediated by Fas cross-linking in NOD and wild-type mice.

**Principal Findings:**

Both effector (CD25^−^, FoxP3^−^) and suppressor (CD25^+^, FoxP3^+^) CD4^+^ T cells are negatively regulated by Fas cross-linking in mixed splenocyte populations of NOD, wild type mice and FoxP3-GFP tranegenes. Proliferation rates and sensitivity to Fas cross-linking are dissociated in Treg cells: fast cycling induced by IL-2 and CD3/CD28 stimulation improve Treg resistance to Fas-ligand (FasL) in both strains. The effector and suppressor CD4^+^ subsets display balanced sensitivity to negative regulation under baseline conditions, IL-2 and CD3/CD28 stimulation, indicating that stimulation does not perturb immune homeostasis in NOD mice. Effective autocrine apoptosis of diabetogenic cells was evident from delayed onset and reduced incidence of adoptive disease transfer into NOD.SCID by CD4^+^CD25^−^ T cells decorated with FasL protein. Treg resistant to Fas-mediated apoptosis retain suppressive activity *in vitro*. The only detectable differential response was reduced Teff proliferation and upregulation of CD25 following CD3-activation in NOD mice.

**Conclusion:**

These data document negative regulation of effector and suppressor cells by Fas cross-linking and dissociation between sensitivity to apoptosis and proliferation in stimulated Treg. There is no evidence that perturbed AICD in NOD mice initiates or promotes autoimmune insulitis.

## Introduction

Eruption of autoimmune insulitis in NOD mice has been attributed to multiple aberrations of immune homeostasis [1], [2], including suboptimal control of effector cells (Teff) [3]–[5], deficient activity of regulatory T cells (Treg) [6]–[10] and dysfunctional interaction between these subsets [10]–[13]. Within the multiple physiological mechanisms of immune homeostasis, negative regulation of expanding clones is a dominant factor in determination of the cellular composition of inflammatory infiltrates. This homeostatic mechanism relies on activation induced cell death (AICD) mediated primarily by the Fas/Fas-ligand (FasL) interaction as the common executioner of apoptosis within the tumor necrosis factor (TNF) superfamily [14]. The relative sensitivity of naïve/effector and suppressor T cells to AICD-type negative regulation has been evaluated as a possible cause of immune dysfunction in type 1 diabetes. T cells isolated from NOD mice are generally more resilient to AICD, including both CD4^+^ [15] and CD8^+^ T subsets [16], a characteristic that becomes more accentuated with age [17]. Similar insensitivity to spontaneous and Fas-mediated apoptosis has been observed in thymocytes of the NOD mice [18], suggesting an inherent deficit in negative regulation of effector T cells. These data led to the concept that decreased susceptibility of pathogenic cells to AICD-type regulation predisposes NOD mice to autoimmune insulitis and affects disease progression.

Addressing the question how differential sensitivities of diabetogenic and regulatory cells to apoptosis affects the evolution of autoimmunity, conflicting evidence has been reported in NOD mice and humans, ranging from resistance [19]–[21] to excessive susceptibility [22]–[24] of Treg to AICD. Most studies have used isolated cell populations disregarding the significant impact of reciprocal interactions between effector and suppressor T cells on sensitivity to apoptosis [19], [25]. For example, IL-2 supplied by effector cells is essential to sustain the suppressive activity of Treg [26] and TCR-associated stimulation protects putative Treg from apoptosis [19], [24]. Consistent wit the observation that CD25^+^ cells are sensitized to FasL in mixed cultures of isolated subsets [19], we have recently demonstrated that the isolation process dominates the sensitivity to spontaneous apoptosis, which deviates substantially from the behavior in mixed cultures [27]. In this study we address two questions: a) whether NOD mice display intrinsic variations in susceptibility to AICD that might contribute to evolution of the autoimmune reaction, and b) what are the relative responses of effector and suppressor CD4^+^ T cells to AICD under stimulation, considering that the patterns of cell death are modulated under continuously changing inflammatory environments [28]. We found no significant differences in Fas-mediated apoptosis between wild type and NOD mice that suggest participation of aberrant negative regulation in evolution of autoimmune insulitis. Although IL-2, TCR-associated activation and costimulation modulate the sensitivity of both effector and suppressor subsets to apoptosis, there is no evidence of perturbed AICD homeostasis in mice with ongoing autoimmune insulitis.

## Results

### Sensitivity of naïve/effector CD4^+^ subsets to Fas cross-linking

**Figure 1:**
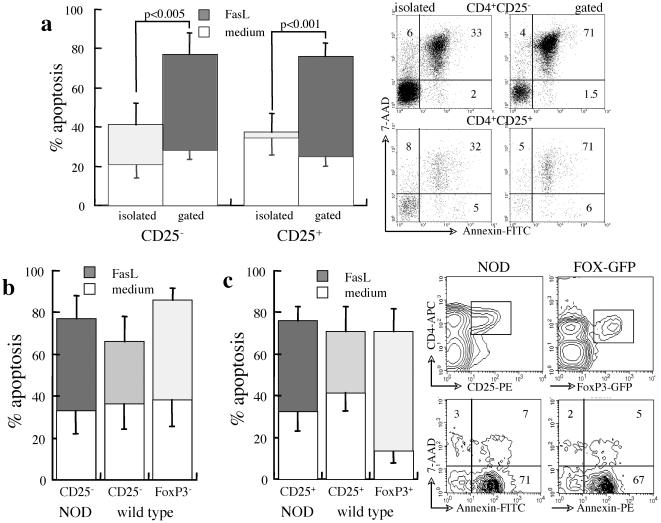
Sensitivity of naïve/effector CD4^**+**^ T to Fas-mediated apoptosis. A. Apoptosis of splenocytes from prediabetic NOD females (14 weeks) during 48 hours of incubation in control medium and induced by 50 µg/ml FasL. Isolated CD4^+^CD25^−^ and CD4^+^CD25^+^ T cell subsets (n = 5) are compared to measurements performed by gating in mixed splenocyte cultures (n = 7). Representative measurements of apoptosis and death assessed by incorporation of Annexin-V and 7-AAD respectively, in isolated and gated CD4^+^ T cell subsets. B–C. Apoptosis of gated naïve/effector CD25^−^ and FoxP3^−^ subsets (B) and CD25^+^ and FoxP3^+^ Treg (C) in mixed cultures of splenocytes from prediabetic NOD females (n = 5), wild type mice (C57/BL, n = 7) and Foxp3-GFP transgenes (n = 5) during 48 hours of incubation in control medium and with 50 µg/ml FasL. The plots of present apoptosis in gated CD4^+^CD25^+^ and CD4^+^FoxP3-GFP^+^ cells.

Similar to high rates of spontaneous apoptosis in cultures of isolated CD25^+^ T cells from wild type mice [19], we have recently documented excessive susceptibility to spontaneous apoptosis of CD25^+^ T cells isolated from diabetic NOD mice, which is reversed by cytokines and cell-to-cell interactions in mixed splenocyte cultures [27]. In variance from comparable levels of spontaneous apoptosis, the composition of the culture affects the sensitivity of various CD4^+^ T cell subsets to FasL: apoptosis measured by differential gating in mixed splenocyte cultures exceeds by far the sensitivity to FasL recorded in isolated subsets (p<0.005, Figure 1A). Although such arbitrary culture conditions are far from simulating the microenvironment in which Treg operate at the site of inflammation [28], mixed cultures appear to better reflect the sensitivity to negative regulation than purified cell subsets.

It has been suggested that relative resistance of naïve/effector T cells to Fas cross-linking affects the evolution of the autoimmune reaction in NOD mice [15]–[17]. Comparative analysis of apoptosis in gated subsets within mixed populations of splenocytes and lymph node cells shows similar sensitivity of CD25^−^ T cells from NOD mice to spontaneous and Fas-mediated apoptosis as the CD25^−^ and FoxP3^−^ subsets in wild type mice (Figure 1B), suggesting that evolution of inflammatory insulitis is not caused by intrinsic deficits in AICD. Likewise, CD25^+^ and FoxP3^+^ T cells in wild type mice display similar high sensitivities to Fas cross-linking (Figure 1C), indicating that regulatory subsets are submitted to AICD-type negative regulation. The similar levels of apoptosis of CD25^+^ T cells from NOD mice indicate that, like effector cells, there is no apparent difference in negative regulation. Taken together, these data show that both effector and suppressor T cells are equally submitted to AICD-type negative regulation in wild type and NOD mice.

### Treg sensitivity to apoptosis under IL-2 stimulation

**Figure 2:**
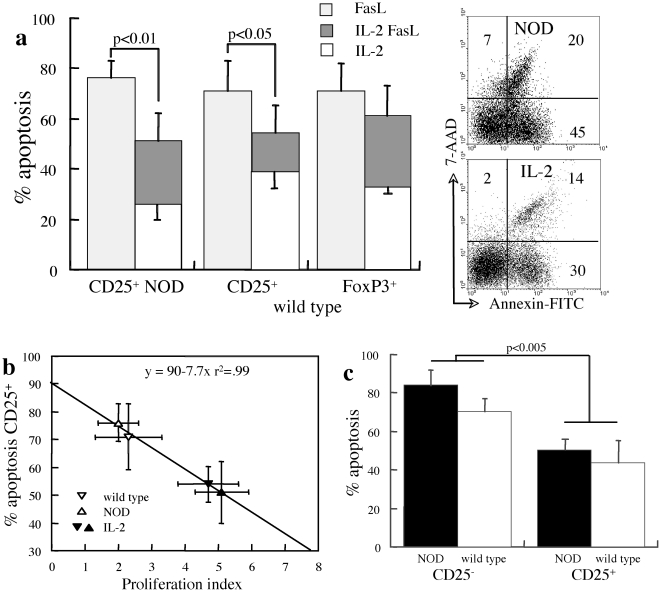
Impact of IL-2 on CD25^**+**^ T cell sensitivity to apoptosis and proliferation. A. Apoptosis of gated CD4^+^CD25^+^ subsets in NOD (n = 5) and wild type mice (n = 7) and CD4^+^FoxP3^+^ T cells in wild type mice (n = 6) during 48 hours of incubation with supplementation of 2000 U/ml IL-2 (IL-2) and with 50 µg/ml FasL (IL-2 FasL) as compared to apoptosis in response to FasL in control medium (FasL). Demonstrative measurements of apoptosis and death assessed by incorporation of Annexin-V and 7-AAD respectively. B. Fas-mediated apoptosis of CD4^+^CD25^+^ T cells from NOD and wild type mice as a function of proliferation rates measured from CFSE dilution, with (n = 4–5) and without (n = 5–7) exogenous supplementation of IL-2 (n = 4–5). C. Apoptosis of gated CD4^+^CD25^−^ and CD4^+^CD25^+^ subsets induced by Fas cross-linking under mitogenic stimulation with ConA in NOD (n = 4) and wild type mice (n = 5).

IL-2 is a significant cytokine involved in amplification of cytotoxic T cell activity and is pivotal to the development and function of Treg, which modulates the sensitivity of lymphocytes to Fas-dependent and Fas-independent apoptosis [28]. The significance of Treg in sustaining self-tolerance and their dependence on IL-2 has been demonstrated by eruption of inflammatory insulitis upon IL-2 neutralization [29] and consistently, a beneficial effect of IL-2 administration over disease progression [30]. In general, naïve/effector T cells supply this cytokine and Treg consume it avidly, creating a cycle of reciprocal dependence on IL-2: Treg inhibit cytokine production limiting the activity of cytotoxic T cells, and in turn IL-2 deficiency downsizes the activity of Treg [26]. Whereas exogenous IL-2 does not affect significantly Fas-mediated apoptosis of CD25^−^ and FoxP3^−^ T cells, exposure of mixed cultures to 2000 U/ml IL-2 decreases Treg apoptosis in NOD and wild type CD25^+^ T cells (Figure 2A). Notably, spontaneous and Fas-mediated apoptosis under the influence of IL-2 is comparable in wild type and NOD mice. IL-2 stimulates faster cycling rates in CD25^+^ T cells from NOD mice (Figure 2B), possibly because the relative IL-2 insufficiency in these mice *in vivo* [30]. The correlation between proliferation and increased susceptibility to Fas-mediated apoptosis is basic feature of AICD in naïve/effector CD25^−^ T cells [31]–[33]. In variance, mitogenic stimulation with ConA shows dissociation between proliferation and sensitivity to Fas-mediated apoptosis in CD25^+^ Treg (Figure 2C), indicating that decreased apoptosis under IL-2 stimulation was partially caused by robust expansion of viable cells.

### Fas cross-linking does not abolish the suppressive activity of CD25^+^ T cells

**Figure 3:**
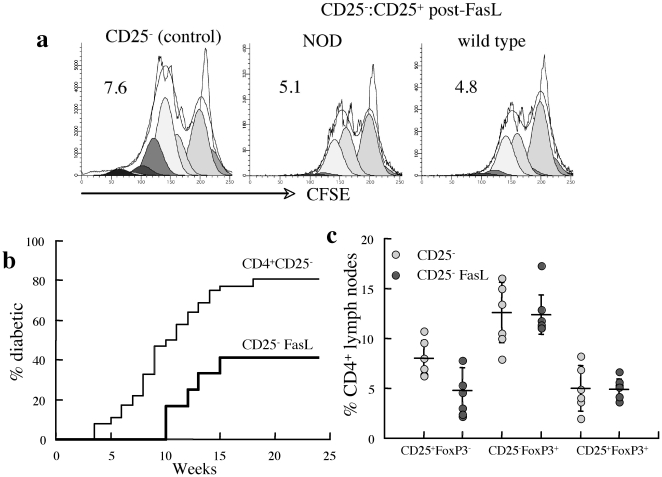
Impact of FasL on effector and regulatory T cell activity. A. Inhibition of proliferation of CFSE-labeled CD25^−^ T cell responders activated with CD3/CD28 (control) by CD25^+^ T cells isolated from NOD and wild type mice after incubation with FasL at a Teff∶Treg ratio of 3∶1. Data are representative of four experiments in which CD25^+^ suppressor cells were co-incubated with CFSE-labeled CD25^−^ responders from the same strain. B. NOD.SCID mice were adoptively transferred with 2.5×10^7^ naïve (n = 36) and FasL-coated CD25^−^ T cells (n = 12) from prediabetic NOD females. Blood glucose was monitored to determine onset of diabetes at levels >200 mg/dl. C. Fractional expression of CD25 and FoxP3 in CD4^+^ T cells from mesenteric/pancreatic lymph nodes of NOD.SCID mice reconstituted with naïve (n = 6) and FasL-coated (n = 5) CD25^−^ T cells from prediabetic NOD females.

We have recently reported that adoptive transfer of CD25^+^ T cells overexpressing FasL protein delays onset and reduces incidence of overt hyperglycemia in prediabetic NOD mice [34]. The current data indicate high sensitivity of these cells to Fas cross-linking during extended *in vitro* culture, questioning the impact of Fas cross linking on the suppressive activity of this subset. To determine how AICD affects the suppressor activity, isolated CD25^+^ T cells were exposed to FasL for 48 hours prior to conincubation with strain-matched CFSE-labeled CD25^−^ T cells under CD/CD28 stimulation. Notably, CD25^−^ T cells display similar responses to CD3/CD28 stimulation in NOD and wild type mice (vide infra). Viable CD25^+^ T cells from wild type and NOD mice had similar suppressive effects on the proliferation of stimulated responders from the respective strains (Figure 3A), emphasizing sustained regulatory activity of CD25^+^ T cells that survive the FasL challenge.

### Autocrine apoptosis reduces the diabetogenic activity of effector T cells *in vivo*

To assess the physiological significance of effector T cell sensitivity to apoptosis, FasL chimeric protein was adsorbed on the surface of CD25^−^ T cells adoptively transferred into NOD.SCID mice. Infusion of FasL-coated CD4^+^CD25^−^ T cells decreased the efficacy of disease transfer and delayed the mean onset time (MOT) to 12±1.9 weeks as compared to 9.5±3.6 weeks in recipients of naïve CD25^−^ cells (p<0.05, Figure 3B). Reduced disease incidence was accompanied by a corresponding decrease in inflammatory islet score, indicating that the diabetogenic potential is reduced by Fas cross linking *in vivo* as previously demonstrated for exposure to FasL *ex vivo* [21], [35]. These data also demonstrate limited toxicity of ectopic FasL protein to the islets [34], an apoptotic pathway that is largely dispensable in the process of destructive autoimmune insulitis [36]. Despite the marked variations in disease onset and incidence, the similar CD4^+^ profiles of mesenteric/pancreatic lymph nodes of NOD.SCID recipients of naïve and FasL-coated CD25^−^ T cells (Figure 3C) suggests that the apoptotic pathways affects primarily the islet reactive cells. Similar delay and reduced incidence of the disease has been observed when CD25^−^ T cells were co-adoptively transferred with FasL-coated CD25^+^ Treg into NOD.SCID mice [34]. Altogether these data underlie the flexibility of CD25^−^ T cells in repopulating NOD.SCID mice to reinstate immune homeostasis in NOD.SCID mice through generation of regulatory subsets.

### Sensitivity to apoptosis under TCR-associated stimulation and costimulation

**Figure 4:**
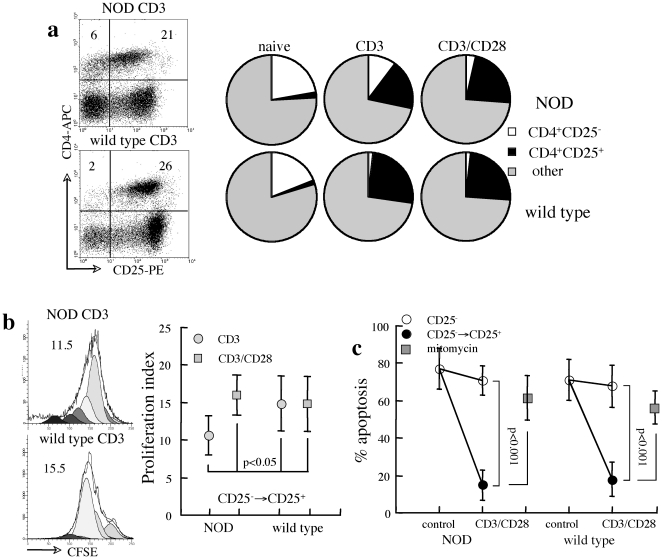
Sensitivity to apoptosis under CD3 and CD3/CD28 stimulation. A. Representative plots of CD25 upregulation under CD3 stimulation in splenocytes from NOD and wild type mice. Quantitative conversion to express CD25 in splenocyte cultures from NOD (n = 4) and wild type females (n = 6) under CD3 and CD3/CD28 stimulation as compared to unstimulated cultures (naïve). Other delineates all CD4^−^ cells. B. Proliferation rates of CD4^+^ T cells that upregulated CD25 expression (CD25^−^→CD25^+^) as determined from CFSE dilution in mixed cultures of splenocytes from NOD (n-4) and wild type mice (n = 5) under CD3 and CD3/CD28 stimulation. C. FasL-induced apoptosis in reference to CD25 expression in gated CD4^+^ T cells within mixed splenocyte cultures from NOD and wild type mice (n = 4) under CD3/CD28 stimulation. Apoptosis was also measured under inhibition of proliferation with Mitomycin C (n = 3).

In next stage we considered that differential susceptibility of effector T cells in NOD mice might be restricted to conditions of stimulation under inflammatory environments. To assess the sensitivity to Fas-mediated apoptosis, CD4^+^CD25^−^ and CD4^+^FoxP3^−^ effector T cells were further characterized under CD3 and CD28 stimulation, which induces robust proliferation and upregulates CD25 expression (Figure 4A). In order to measure apoptosis in mixed cultures, we considered that upregulation of CD25 in the majority of CD25^−^ T cells (CD25^−^→CD25^+^) dominates the insignificant minor fraction (<10%) of naturally occurring CD25^+^ T cells. Comparative analysis reveals reduced responsiveness of NOD lymphocytes to CD3 stimulation, including both upregulation of CD25 (p<0.01, Figure 4A) and proliferation (p<0.05, Figure 4B), which is compensated by additional CD28 costimulation. Reduced proliferation rates of NOD lymphocytes under CD3 activation suggest lesser responsiveness to TCR-associated stimulation due to a higher intrinsic state of activation associated with autoimmune inflammation. Fast cycling rates of CD25^−^ T cells that upregulate CD25 expression under CD3 and CD3/CD28 stimulation reduce fractional apoptosis, whereas cells with sustained CD25^−^ phenotype display high levels of FasL-induced apoptosis (Figure 4C). Reduced levels of apoptosis caused by fast cycling of viable cells was confirmed by inhibition of cell proliferation with Mitomycin C, which increased the fractions of apoptotic cells. Importantly, the similar sensitivities to Fas cross-linking demonstrate that TCR-associated activation and costimulation do not perturb a balanced negative regulation of the naïve/effector lymphocytes in NOD mice.

**Figure 5:**
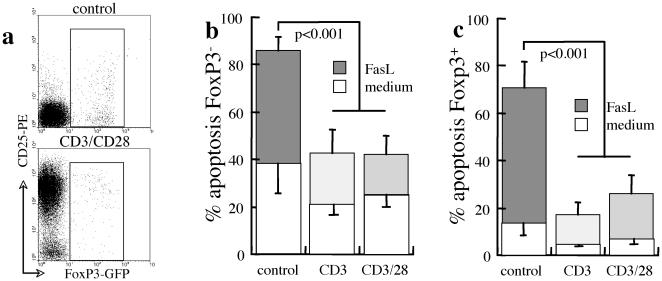
Sensitivity to apoptosis under stimulation in reference to FoxP3 expression. A. CD4^+^ T cells do not upregulated FoxP3 expression under CD3/CD28 stimulation in mixed splenocyte cultures from FoxP3-GFP transgenes. Apoptosis was determined in control medium and with FasL under CD3 and CD3/CD28 stimulation in (B) gated FoxP3^−^ T cells (n = 5) and (C) gated FoxP3^+^ T cells (n = 6) in mixed cultures of splenocytes from transgenic FoxP3-GFP mice.

In variance from CD25 upregulation, FoxP3 is not induced by CD3 and CD28 stimulation, allowing measurements of apoptosis of CD4^+^FoxP3^−^ and CD4^+^FoxP3^+^ T cells in mixed cultures of splencoytes from FoxP3-GFP transgenes (Figure 5A). Both FoxP3^−^ effector (Figure 5B) and FoxP3^+^ Treg subsets (Figure 5C) display reduced susceptibility to spontaneous apoptosis and sensitivity to Fas cross-linking under CD3 and CD3/CD28 stimulation, indicating sustained viability by TCR-associated activation and costimulation. Under such conditions the Treg subset appears to benefit of superior viability (p<0.01), consistent with activation of suppressor mechanisms in early stages of immune activation by reduced sensitivity to AICD-type negative regulation.

## Discussion

Among multiple pathways of immune deregulation, anomalies of T cell sensitivity to apoptosis are considered to contribute to eruption of diabetes in NOD mice [15]–[17]. The proposed scenario of perturbed immune homeostasis in diabetes-prone mice and humans includes resistance of effector cells to apoptosis [3]–[5], in part due to deficient expression of the Fas receptor [37], and increased susceptibility of suppressor cells to apoptotic death [38]–[40]. In this study we provide evidence that Treg are submitted to clonal deletion by Fas cross-linking, and peripheral AICD negative regulation of T cells is largely intact in NOD mice in advanced stages of inflammatory insulitis. Therefore, neither resistance of effector cells nor sensitivity of regulatory cells to AICD-type negative regulation predispose to evolution of autoimmune insulitis.

Treg are extremely sensitive to their environment. These cells operate primarily at the site of inflammation within a dynamic environment influenced by cytotoxic T cells as well as tissue injury [28], including a series of feedback mechanisms that modulate their activity [41]–[44]. Therefore, assessment of sensitivities to spontaneous and Fas-mediated apoptosis in suspensions of purified T cell subsets is unlikely to reflect a true behavior [19], [27]. For example, proliferative anergy characteristic of purified Treg *in vitro* is in sharp contrast from the faster proliferation rates of these cells *in vivo* [24], [25], [43], [44]. Likewise, purified CD25^+^ cells display excessive sensitivity to spontaneous apoptosis in culture [20], [22]–[24], which is common to wild type, prediabetic and diabetic NOD females [27]. Considering mixed cultures as a better model to assess AICD [19], [25], [27], it is evident that both anti- and pro-apoptotic signals originate from adjacent cells. On the one hand, increased spontaneous apoptosis of CD25^+^ T cells is reversed in mixed cultures [27], underlining the provision of anti-apoptotic factors and signals by other cell subsets. On the other hand, apoptosis triggered by Fas cross-linking is amplified in mixed cultures, disclosing the presence of proapoptotic factors that augment AICD-type negative regulation in both subsets. Here we emphasize that Fas cross-linking is a homeostatic mechanism of negative regulation of naturally occurring Treg.

Cytokine deprivation has profound effects on the sensitivities to apoptosis of both effector [45] and suppressor subsets [46]. IL-2 is a major cytokine associated with cell viability, as demonstrated by the reduced intrinsic sensitivity to apoptosis of lymphocytes deficient in IL-2 and its cognate receptors [31]–[33], [47], [48]. Naturally occurring Treg are avid consumers of IL-2 but do not produce this cytokine [49], which modulates both effector and suppressor activities [50]. In variance from correlated cycling and sensitization to apoptosis of CD25^−^ naïve/effector T cells by IL-2 [31]–[33], proliferation and sensitivity to FasL are dissociated in CD25^+^ Treg: IL-2 causes dilution of dead cells due to robust expansion of viable cells. Uncoupling between proliferation and Fas-dependent negative regulation evolves as a particular Treg characteristic, which maintains viability of this subset with intrinsic state of activated suppressor activity under steady state conditions [28], [44]. Furthermore, TCR-associated stimulation independently protects Treg from apoptosis, exceeding its effect on naïve/effector cells. Considering that TCR engagement is essential to initiate Treg cycling under IL-2 stimulation [26], [51], both stimulatory pathways are shown to support the function and viability of regulatory T cells.

Naïve/effector cells from wild type and NOD mice display similar sensitivities to Fas cross-linking under TCR-associated activation and CD28 costimulation in mixed cultures, including the subset induced into fast proliferation that upregulates CD25 expression. Our data corroborate the observation that sensitivity to apoptosis under CD28 costimulation is not entirely dependent on Fas cross-linking [19], [52] and demonstrates the reciprocal condition where this apoptotic pathway operates independent of costimulation. Whereas CD28 costimulation superposed on CD3 activation increases the susceptibility of naïve/effector T cells to spontaneous apoptosis [27], it does not sensitize these cells to Fas cross-linking. Thus, exacerbation of diabetes by CD28 deficiency in NOD mice [52] and the reciprocal protection by CD28 costimulation in neonatal NOD [53] and T cell depletion with anti-CD3 antibodies [54] point to Treg as the primary mechanism responsible for immunomodulation.

Among multiple mechanisms of immune deregulation in NOD mice, there is no evidence of defective peripheral negative regulation and dysfunctional AICD as predisposing factors in evolution of inflammatory insulitis. Inasmuch as immune deregulation is attributed to aberrant sensitivity to Treg-mediated suppression of effector cells in NOD mice [3]–[5] and humans [55], our data present evidence of protracted negative regulation of naïve/effector CD4^+^ T cells under stimulatory conditions. Balanced AICD-type negative regulation of effector and suppressor cells in wild type and NOD mice is consistent with similar distribution of CD4^+^ subsets under steady state conditions as well as in reconstituted NOD.SCID mice. The diabetogenic potential is impaired by overexpression of FasL on CD25^−^ cells, similar to the inhibitory activity of this apoptotic pathway in lymphocytes of diabetic NOD mice *in vitro* [35]. Whereas the Fas receptor is evenly expressed by effector and suppressor subsets, exclusive expression of the cognate ligand in CD25^−^ cells suggests auocrine and paracrine (fratricide) negative regulation of diabetogenic and suppressor clones within the inflammatory infiltrates and regional lymphatics [21]. Notably, FasL is dispensable in the process of destructive inflammatory insulitis [36] and does not impose severe threat to naïve islets from NOD and wild type mice [34], [56] unless expressed as a transgene in β cells [57], therefore it can be used for immunomodulation in both transplant and autoimmune settings [14], [34], [58].

A significant difference in the naïve/effector subset of T cells is the slower proliferation in response to mitogenic CD3 ligation in NOD mice, which is restored by CD28 costimulation. Effector cells in NOD mice are also less sensitive to radiation [59], resulting in disease recurrence upon recovery from immunosuppression [60], enhanced bone marrow allograft rejection [61] and persistent autoimmune insulitis even in the absence of alloreactivity [59], [62]. In the transplant setting, perturbed homeostasis during recovery from conditioning-induced lymphopenia contributes to disease recurrence [63] unless negative regulation is restored by mixed allogeneic chimerism [64]. In addition, the effect of IL-2 on Treg in NOD mice appears to be more pronounced, consistent with an intrinsic state of activation concurrent with the ongoing inflammatory state [28] that is characterized by relative IL-2 insufficiency [30]. Although IL-2 is a significant factor in sustaining Treg resistance to apoptosis, as also demonstrated in isolated CD25^+^ T cell suspensions [27], this cytokine and its relative deficiency do not cause aberrant clonal deletion in NOD mice. On the contrary, support of Treg viability is an apparent mechanism that participates in amelioration of inflammatory insulitis by IL-2 administration [30], [65].

In summary, we demonstrate: a) Treg are submitted to AICD-type regulation mediated by Fas cross-linking, b) proliferation and sensitivity to Fas-mediated AICD are dissociated in Treg, c) effective clonal deletion of both subsets under stimulatory conditions, d) balanced sensitivity to negative regulation of effector and suppressor CD4^+^ T cells, and e) insignificant differences in AICD-type negative regulation between wild type and NOD mice. Altogether these data indicate that negative regulation though apoptosis is not a major factor that predisposes to immune deregulation and evolution of autoimmune insulitis in NOD mice.

## Materials and Methods

### Animals

Mice used in this study were C57BL/6 (wild type), non-obese diabetic (NOD) and B6.Cg-Foxp3^tm2Tch^ transgenic mice (expressing GFP under control of the FoxP3 promoter). Animals purchased from Jackson Laboratories (Bar Harbor, ME) were inbred and housed in a barrier facility. The Institutional Animal Care Committee of the Schneider Medical Center has approved all procedures #022B6229 dated 4.1.2009. Blood glucose was monitored between 9–11 AM in tail blood samples at weekly intervals using a glucometer (Accu-Chek Sensor, Roche Diagnostics, USA). Diabetes was defined as two consecutive blood glucose measurements above 200 mg/dl [59].

### Isolation of cells according to CD25 expression

Cell suspensions from spleens and lymph nodes were prepared as previously reported [5], [59]. CD25^−^ and CD25^+^ subsets of CD4^+^ T cells are isolated from the spleens and mesenteric lymph nodes using the CD4^+^CD25^+^ Regulatory T cell isolation kit (Miltenyi Biotec, Bergisch-Gladbach, Germany) [5]. Briefly, CD4^+^ T cells are negatively selected and positive CD25^+^ selection was performed using PE-labeled monoclonal antibodies conjugated to anti-PE magnetic microbeads, which were retained during passage through a magnetic field. Immunomagnetic isolation of cells from NOD females yields a CD4^+^CD25^−^ subset contaminated with 0.7±0.4% and 3.6±1.3% CD25^+^ and FoxP3^+^ cells respectively, and a CD4^+^CD25^+^ subset of which 75±4% expressed FoxP3, and are contaminated with 17±8% CD25^−/low^ cells.

### Flow cytometry

Cells composition was determined using antibodies conjugated to fluorescein isothyocyanite (FITC), phycoerythrin (PE), allophycocyanin (APC) and peridinin chlorophyll a-protein (PerCP, BD Pharmingen, San Diego, CA): CD4 (clone RM 4-5), CD8 (clone 53-6.7), CD25 (clone PC61.5) [5], [27]. FoxP3 was determined following permeabilization and intracellular staining with a PE-labeled antibody (Foxp3 staining buffer set NRRF-30, eBioscience, San Diego, CA). Antibodies were purchased from BD Pharmingen and eBioscience. Cell death and apoptosis were determined in cells incubated with 5 µg/ml 7-aminoactinomycin-D (7-AAD, Sigma, St. Lois, MO) and Annexin-V (IQ products, Groningen, The Netherlands), respectively. Measurements were performed with a Vantage SE flow cytometer (Becton Dickinson, Franklin Lakes, NJ). Positive staining was determined on a log scale, normalized with control cells stained with isotype control antibodies.

### *In vitro* apoptosis

2×10^6^ cells/ml were incubated in DMEM supplemented with 2 mM L-glutamine, 1 mM sodium pyruvate, 13.6 µM folic acid, 270 µM L-asparagine, 548 µM L-arginine HCL, 10 mM HEPES, 50 µM 2β-Mercaptoethanol, 100 mg/ml streptomycin, 100 U/ml penicillin and 5% heat-inactivated fetal bovine serum (FBS) (MLR medium) [60]. All ingredients were purchased from Beit Haemek and Sigma (St. Lois, MO). Apoptotic challenge was applied by addition of 50 µg/ml FasL chimeric protein. Cells were stimulated by exogenous supplementation of 2000 U/ml IL-2 (Peprotech, London, UK), anti-CD3 (R&D Systems, Minneapolis, MN) and beads conjugated to anti-CD3 and anti-CD28 (Invitrogen, Oslo, Norway) at a bead∶cell ratio of 1∶1. For determination of apoptosis in reference to FoxP3 expression, cells were first exposed to Annexin-V and subsequently were stained for FoxP3 expression.

### Proliferation assay

Splenocytes and lymphocytes were incubated at room temperature for 7 minutes with 10 µM 5-(and 6-)-carboxyfluorescein diacetate succinimidyl ester (CFSE, Molecular Probes, Carlsbad, CA) [27]. The labeled responders were cultured at 37°C in a humidified 5% CO_2_ atmosphere for 2 days in MLR medium containing 5% heat-inactivated mouse serum and 20 U/ml IL-2. All proliferation assays were performed in triplicates. Suppression of proliferation of CFSE-labeled responders stimulated with CD3/CD28 was performed by co-culture with isolated CD25^+^ T cells at various Teff∶Treg ratios for 48 hours. CFSE dilution was analyzed in flow cytometry by gating on the live lymphocytes and proliferation was quantified with ModFit software (Verity Software House, Topsham, ME).

### Adsorption of FasL protein on cell surface

Splenocytes harvested under aseptic conditions were suspended in 5 µM freshly prepared EZ-Link Sulfo-NHS-LC-Biotin (Pierce) in PBS for 30 minutes at room temperature [66]. Washed cells were incubated with streptavidin-FasL chimeric protein (100 ng protein/1×10^6^ cells in PBS). After two additional washes the efficiency of adsorption was evaluated by flow cytometry using anti-streptavidin and anti-FasL antibodies.

### Histology

Pancreata were excised from mice euthanized by CO_2_ asphyxiation, and were fixed in ice-cold PBS containing 1.5% fresh paraformaldehyde for 2 hours at 0–4°C before overnight immersion in 30% sucrose [59]. Tissues were embedded in OCT (Sakura Finetek, Torrance, CA), frozen in isopentane suspended in liquid nitrogen, sectioned (3–6 µm) with a Cryotome (Thermo Shandon, Cheshire, UK) and stained with hematoxylin and eosin. Islet inflammation was scored according to: 0-no inflammation, 1-peri-insulitis, 2-inflammatory infiltration <50% of islet area, 3-inflammation >50% of islet area and 4- disruption of islet structure.

### Statistical analysis

Data are presented as means ± standard deviations for each experimental protocol. Results in each experimental group were evaluated for reproducibility by linear regression of duplicate measurements. Differences between the experimental protocols were estimated with a post hoc Scheffe *t*-test and significance was considered at p<0.05.
